# Health-Related Quality of Life Changes in Patients with Digestive Cancers and Chronic Digestive Diseases: A Prospective, Multicenter Study

**DOI:** 10.3390/jcm15103596

**Published:** 2026-05-08

**Authors:** Josune Martín, Nere Larrea, Yolanda García, Inmaculada Bolinaga, Amaia Perales, Cristina Sarasqueta, Ana González-Pinto, José M. Quintana

**Affiliations:** 1 Department of Neuroscience, University of the Basque Country UPV/EHU, 48940 Leioa, Vizcaya, Spain; 2 Research Unit, Galdakao-Usansolo University Hospital, Osakidetza (Basque Health Service), Barrio Labeaga s/n, 48960 Galdakao, Bizkaia, Spain; nlarrea.biosistemak@gmail.com (N.L.); jmqlai01@gmail.com (J.M.Q.); 3 Biosistemak Institute for Health Systems Research, 48001 Barakaldo, Bizkaia, Spain; 4 Research Network on Chronicity, Primary Care and Prevention and Health Promotion (RICAPPS), 48960 Galdakao, Bizkaia, Spain; cristina.sarasquetaeizaguirre@osakidetza.eus; 5 Department of Endocrinology, Galdakao-Usansolo University Hospital, Osakidetza (Basque Health Service), Barrio Labeaga s/n, 48960 Galdakao, Bizkaia, Spain; mariayolanda.garciafernandez@osakidetza.eus (Y.G.); ibolinaga@hotmail.com (I.B.); 6 BioBizkaia Health Institute, 48003 Barakaldo, Bizkaia, Spain; 7 Biodonostia Health Institute, 20014 San Sebastián, Gipuzkoa, Spain; amaia.peralesanton@bio-gipuzkoa.eus; 8 Donostia Hospital, 20014 San Sebastián, Gipuzkoa, Spain; 9 Department of Psychiatry, Araba University Hospital, Osakidetza (Basque Health Service), 01009 Vitoria-Gasteiz, Spain; anamaria.gonzalezpintoarrillaga@osakidetza.eus; 10 Bioaraba Health Research Institute, 01009 Vitoria-Gasteiz, Álava, Spain; 11 Health Management Unit, Araba University Hospital, Osakidetza (Basque Health Service), 01009 Vitoria-Gasteiz, Spain; 12 Mental Health Networking Biomedical Research Centre (CIBERSAM), 01009 Vitoria-Gasteiz, Spain

**Keywords:** GLIM criteria, malnutrition, persons with cancer, persons with digestive diseases, health-related quality of life

## Abstract

**Background**: This study sought to appraise health-related quality of life (HRQoL) one year after admission in persons with digestive cancer and chronic digestive diseases, stratified by nutritional status on hospital admission, and to identify clinical predictors of HRQoL. **Methods**: HRQoL questionnaires were completed on admission and after 12 months by a prospective cohort of 560 persons with digestive cancers and digestive chronic diseases. Between 2020 and 2021, among the 926 patients admitted to the study, a prospective cohort of 560 persons with digestive cancers or digestive chronic diseases completed HRQoL questionnaires at admission and after 12 months. Nutritional status was assessed using MUST and GLIM criteria. Multiple correspondence analyses were used to identify clinically meaningful subgroups of patients. Multivariate analyses were performed to identify HRQoL predictors. **Results**: Participants had a mean age of 63.4 years; 60.7% were male and 39.3% female. At baseline, 34.81% of participants were classified as malnourished. Scores for mental health (SF-36 MH), role-emotional (SF-36 ER), and vitality (SF-36 VT) were significantly lower among persons with severe malnutrition and colorectal cancer (CRC) (*p* = 0.001, *p* = 0.004 and *p* = 0.009, respectively). Regarding changes in HRQoL over one year, persons with severe malnutrition at baseline showed the greatest improvement in mental health (SF-36 MH) within the CRC group (*p* = 0.01). Among persons with cancer, polypharmacy was consistently associated with poorer outcomes, with significant negative associations across EuroQoL (*p* < 0.001), SF-36 Social Functioning (*p* < 0.001), and General Health (*p* < 0.001). In persons with digestive diseases, length of admission was inversely associated with Physical Role (*p* < 0.05) and Body Pain (*p* < 0.05). Finally, GLIM status displayed limited associations, only significant in the case of Vitality and Body Pain (*p* < 0.05 in both). **Conclusions**: HRQoL in patients with oncological and digestive diseases is shaped by a complex interplay of clinical and sociodemographic factors.

## 1. Introduction

Health-related quality of life (HRQoL) is now a key outcome in the management of persons with chronic diseases, particularly in oncological and digestive pathologies [1]. As survival rates improve, attention has shifted toward understanding the broader impacts of disease and treatment targeting patients’ physical, emotional, and social well-being [2,3]. Among the many factors influencing HRQoL, nutritional status plays a pivotal role, especially in populations with high metabolic demands and treatment-related side effects [4,5].

Malnutrition is a highly prevalent condition among persons with digestive cancer and other gastrointestinal diseases, with estimates ranging from 30% to 80% depending on disease type and stage [6]. In colorectal cancer (CRC), for instance, malnutrition is frequently observed on diagnosis and is associated with increased morbidity, reduced treatment tolerance, and poorer quality of life [7]. Similarly, persons with inflammatory bowel disease (IBD) and pancreatitis often experience nutritional deficits due to malabsorption, inflammation and dietary restrictions [8]. Hospital malnutrition continues to be an often underrecognized issue in Spain, with estimates of prevalence ranging from 24% to 62% [9,10]. While the condition is treatable, it is often insufficiently identified and managed. Hospitalization can negatively impact both HRQoL and functional autonomy, especially in persons with chronic illnesses. In order to guide more effective, patient-centered interventions, it is therefore essential to assess HRQoL post-discharge and identify the variables that shape patients’ long-term health perceptions.

With a view to standardizing the diagnosis of malnutrition, the Global Leadership Initiative on Malnutrition (GLIM) proposed a set of criteria combining phenotypic and etiological components. GLIM has gained traction in clinical nutrition and some studies suggest a correlation between GLIM-defined malnutrition and impaired HRQoL among persons with cancer [11,12] and persons with IBD [13,14]. Despite a growing body of literature, most studies focus on single disease entities or short-term outcomes, limiting our understanding of how nutritional status interacts with other clinical variables to shape long-term HRQoL trajectories.

This study therefore seeks to provide a comprehensive, one-year longitudinal analysis of HRQoL in a diverse cohort of persons with oncological and chronic digestive diseases, stratified by nutritional status at hospital admission. Our study hypothesis is that persons with malnourishment on admission will have worse HRQoL one year later. Additionally, to identify clinically meaningful subgroups among hospitalized patients, multiple correspondence analysis (MCA) was used to clarify the multifactorial nature of HRQoL and identify targets for more effective and personalized care.

## 2. Methods

### 2.1. Study Design and Patient Selection

A prospective cohort study was performed in three Spanish public hospitals on persons with oncological or chronic digestive pathologies between June 2020 and December 2021. The three hospitals were Galdakao-Usansolo University Hospital, which caters to an urban and semi-urban population of about 300,000; Basurto University Hospital, which serves an urban population of around 350,000 in Bilbao; and Donostia University Hospital which caters to an urban and semi-urban population of around 400,000 in the city and environs of San Sebastián/Donostia. The three have similar human and technological resources and serve socio-demographically and clinically analogous populations. The project was evaluated by the three centers’ research committees, all of which gave their approval, as did the Basque Ethics Committee for Clinical Research (CEIC) (Approval Number: PI2018181). It was entered in ClinicalTrials.gov as ID NCT04188990.

***Criteria for inclusion:***

Participants were considered eligible for inclusion provided they met all of the following conditions:

- Aged more than 18 years.
- Had given prior informed consent.
- Met all criteria for inclusion and none for exclusion.
- Had been enlisted for the study in the first 48 h after being admitted to the digestive, oncological or surgical departments of any of the participating centers.
- Presented with one of the following pathologies:
  - Digestive: ulcerative colitis, Crohn’s disease or acute pancreatitis;
  - Oncological: cancer of the bowel, stomach, rectum, pancreas or esophagus.

***Exclusion criteria:***

Persons were excluded if they met any of the following:

- Had undergone surgery for colon or rectum cancers detected via screening.
- Had serious organic or psychopathological problems or were in a terminal stage of their illness.
- Were admitted to critical or short-term care units.
- Were participating in a study on nutritional intervention.
- Were pregnant.
- Had neurosensory issues.
- Were unable to understand Spanish or complete the questionnaires used in the study.
- Had not signed the informed consent form.

### 2.2. Procedure

Recruitment was carried out by the clinical services within the first 48 h of admission, and nutritional assessments were performed by the nutritionists occurred within the first 72 h. Thus, in the 72 h after being admitted to any of the three centers, all patients who fulfilled the criteria for selection were evaluated individually by means of the GLIM criteria and Malnutrition Universal Screening Tool (MUST) [15,16]. The assessment was performed in the endocrinology and nutrition services of the respective hospitals by two trained nutritionists (one attending to the hospitals in Galdakao and Basurto, and another to Donostia Hospital). They performed the screening based on MUST (they conducted nutritional histories, weighed the patients, and evaluated food intake). Based on GLIM criteria, three groups of patients were identified: persons without malnutrition; persons with moderate malnutrition; and persons with severe malnutrition. In all cases, clinical data and outcomes were evaluated when the patients were admitted and exactly 12 months afterwards.

### 2.3. Data Collection and Instruments

#### 2.3.1. Clinical, Anthropometric, Nutritional and Socio-Demographic Data

Upon admission, during hospitalization, and at the 12-month checkpoint, we retrieved a range of information from patients’ clinical records, namely *sociodemographic information on admission*: age, gender; *clinical data on admission and during hospitalization*: primary pathology (cancer of the esophagus, stomach or pancreas, CRC, ulcerative colitis, pancreatitis or Crohn’s disease), admission type (medical or surgical), surgical intervention, comorbidities per the Charlson Comorbidity Index, treatment prescribed, duration of hospitalization, complications related to infection during hospitalization, *clinical data at follow-up*: 90-day readmission, mortality; *nutritional and anthropometric data during hospitalization*: Body Mass Index (BMI), weight, calf diameter and HGS (hand grip strength), measured with a Jamar Hydraulic Hand Dynamometer.

#### 2.3.2. Patient-Reported Measurements

The patients filled out Spanish-language versions of the following:

*Barthel Index*. Assessment of Performance in Basic Activities of Daily Living. The Barthel Index [17] evaluates ability to perform everyday activities, taking into account ten basic functions. It is scored from 0 to 100, with a higher score indicating that the subject is more likely to be able to live at home after discharge from hospital with some independence. Four categories were created using cutoff points one the scale: a reference value of 75–100 (no/mild disability), 50–74 (moderate disability), 25–49 (severe disability), and 0–24 (entirely dependent) [18].

*EQ-5D.* The self-report version of the *EuroQoL generic health-related quality of life questionnaire* [19,20] is structured in two parts: the EQ-5D- 5L descriptive system (Scale I) and the EQ Visual Analogue scale (VAS) (Scale II). In Scale I, participants choose one of five degrees of severity in five different dimensions. Scale II maps their self-rated health on a vertical, 20 cm visual analogue scale, going from 0 (corresponding to the worst imaginable state of health) to 100 (best imaginable state of health).

*SF-36 Health Questionnaire v2*. This generic assessment of HRQoL identifies positive and negative health and looks at both mental and physical health. Participants answer 36 questions in all comparing their current health status to one year ago, addressing 8 dimensions of health: physical function, physical role, body pain, general health, vitality, social function, role-emotional and mental health. It is scored from 0 to 100; the higher the score, the better the health status. The Spanish-language version of the SF-36 has been validated among populations in Spain [21].

#### 2.3.3. Power Analyses

The required sample size was estimated based on the primary objective of detecting differences in HRQoL between patients with and without GLIM-defined malnutrition. Assuming a small-to-moderate standardized effect size (Cohen’s d = 0.30) in a key SF-36 domain as General Health, for a two-sided alpha of 0.05, 80% power, and an allocation ratio of approximately 1:2 (malnourished vs. non-malnourished), the minimum required sample size was estimated at about 350–360 participants with complete follow-up data. Anticipating up to 30% attrition at 12 months, we planned to recruit at least 500 patients at baseline. Ultimately, 926 patients were enrolled, and 560 completed the 12-month assessment, thus exceeding the a priori sample size requirement.

#### 2.3.4. Statistical Analyses

The statistical analyses started with a descriptive analysis. For categorical variables, the sample size (n) and percentage were reported. For continuous variables, either the mean and standard deviation or the median and interquartile range were reported, depending on distribution. We also performed comparisons using Chi-squared test for categorical variables and Kruskal–Wallis test for continuous variables. We then performed multivariable analysis to explore the association between several factors and the different quality-of-life outcomes assessed: EuroQoL, Barthel, and SF-36. For each association, we used linear multilevel models and reported the beta coefficient (β) and its 95% confidence interval (CI). The predictive capability of the models was assessed using the coefficient of determination (R^2^). In addition to the predefined subgroup analyses, we conducted multivariable multilevel linear models including all patients in the cohort to identify predictors of one-year HRQoL and functional outcomes at the population level. To explore whether the effect of these predictors differed between diagnostic categories, we included interaction terms between pathology group (gastrointestinal cancer vs. other digestive diseases) and key clinical variables (gender, age, CCI, days of admission, surgical intervention, and polypharmacy).

To define participant types, multivariate techniques were used. In order to identify cluster associations in the data that might show groups with similar characteristics, we conducted multivariable techniques such as factor analysis (FA), multiple correspondence analysis (MCA), and cluster analysis (CA). We selected MCA as the multivariable technique for the analysis because most of the variables were categorical. Using this technique, the information contained in the original categorical active variables is transformed into continuous factors. These are represented by numerical values and a positive/negative sign, which are used for interpretation. CA classifies information into relatively homogeneous groups based on the values of different variables. The combination of MCA and CA could place subjects into groups suggested by the data—not defined a priori—where subjects in each group are similar but dissimilar to subjects in other groups [22]. The MCA was conducted on 24 categorical variables, all entered as active variables: age category, sex, patient group, hospital, surgical complications, length of stay category, type of admission, surgical intervention, GLIM-based nutritional status, Charlson comorbidity category, need for support, prior chemotherapy, alcohol use, Barthel Index category, EuroQoL category, and the nine categorized SF-36 domains. No supplementary variables were included in the MCA. Dimensions retained for interpretation were the first two MCA dimensions, because they captured most of the adjusted inertia (46.2% and 24.3%, respectively; cumulative 70.5%) and showed the clearest clinical interpretability. Individual coordinates on these two dimensions were then used as input for the clustering procedure. CA was subsequently performed on the individual factor scores from Dimensions 1 and 2 using hierarchical agglomerative clustering with Manhattan distance and Ward’s linkage method. The final partition was obtained by cutting the dendrogram into three clusters. Given the exploratory nature of the analysis, no formal internal validation or stability analysis was performed. Therefore, the identified profiles should be interpreted as hypothesis-generating and require confirmation in external datasets.

We performed all statistical analyses with SAS for Windows, version 9.4 (SAS Institute, Carey, NC, USA) and R^©^, version 4.2.0.

## 3. Results

### 3.1. Description of the Sociodemographic, Clinical, and Anthropometric and Nutritional Data

The 926 individuals in the study sample were recruited from June 2020 to December 2021. All had digestive and oncological pathologies, met the criteria for selection, and filled in the baseline questionnaires. Of the total number, 560 underwent the 12-month follow-up (365 persons without malnutrition and 195 persons moderately or severely malnourished) (Figure S1). A total of 145 patients (15.66%) died prior to the 1-year follow-up. The deceased were older (69 years on average) with a higher proportion of colon/rectal cancer (52.4%), lower BMI (24.2 ± 4.7) at admission, higher readmission at 90 days (46.2%), and scored worse in functional and HRQoL parameters. Those who failed to respond at one year (23.87%) were younger (56.0 ± 16.8) and persons with IBD and pancreatitis diagnoses (57%) (Table S1). The mean age of the sample who responded at follow-up was 63.4 (SD = 16.2) of whom 60.7% were male, and 49.3% had IBD or pancreatitis. Of the 77 persons severely malnourished, 44.2% had CRC, and 19.5% were readmitted at 90 days. Significant differences were observed across malnutrition categories: persons with severe malnutrition were more frequently diagnosed with CRC, other cancer, and IBD, while persons non-malnourished were predominantly affected by pancreatitis (*p* < 0.0001) (Table S2). There were no statistically significant differences between the three GLIM groups in gender, Charlson Index, number of drugs, type of admission, surgical intervention, infectious complications, handgrip strength at admission, nasogastric or nasojejunal nutrition, gastrojejunostomy, and parenteral nutrition (Table 1).

### 3.2. Description of the Baseline, One-Year Follow-Up, and Change from HRQoL of the Participants

No significant differences were observed at baseline for functional status across GLIM groups. However, mental health scores (SF-36 MH) were significantly lower among persons with CRC who suffered from severe malnutrition (*p* = 0.001). Similarly, scores for role-emotional (SF-36 ER) and vitality (SF-36 VT) were significantly lower in this group (*p* = 0.004 and *p* = 0.009, respectively). Social functioning (SF-36 SF) and role-physical (SF-36 PR) were also significantly impaired among persons with other cancers and IBD with severe malnutrition (*p* = 0.03). General health (SF-36 GH) was significantly lower in persons with severely malnourished CRC (*p* = 0.04).

No significant differences were observed in EuroQoL or physical functioning (SF-36 PF) across the malnutrition groups (Table 2). At one-year follow-up, no statistically significant differences were observed across malnutrition categories for functional status and HRQoL (Table 2). Changes in HRQoL over one year varied significantly across malnutrition groups and pathologies. Persons with severe malnutrition at baseline showed the greatest improvement in mental health (SF-36 MH) within the CRC group (*p* = 0.01). Physical functioning (SF-36 PF) improved significantly in persons severely malnourished with other cancers (*p* = 0.005). Social functioning (SF-36 SF) and physical role (SF-36 PR) also showed notable gains among persons severely malnourished with other cancers and IBD (*p* = 0.009 and *p* = 0.03, respectively). Conversely, persons with pancreatitis and severe malnutrition experienced a significant decline in physical role (SF-36 PR) (*p* = 0.0008). Improvements in bodily pain (SF-36 BP) were observed in persons severely malnourished with other cancers (*p* = 0.02). No significant changes were observed in EuroQoL, Barthel Index or General Health (SF-36 GH) across groups (Table 2).

### 3.3. Results of the Multiple Correspondence Analysis

**Figure 1:**
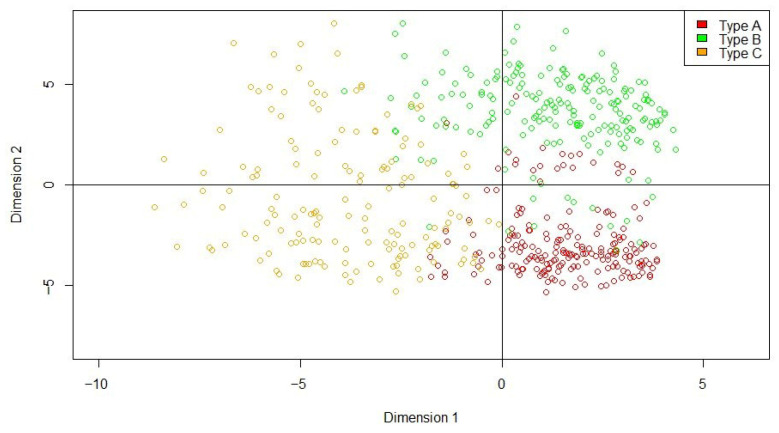
Multiple Correspondence Analysis (MCA)-derived clinical and nutritional profiles among participants with digestive cancers and chronic digestive diseases. Dimension 1: Clinical variables. Dimension 2: Sociodemographic and HRQoL variables. Figure legend: MCA identified three distinct subgroups with differing nutritional status and HRQoL patterns: Type A showed the most favorable outcomes; Type B presented a more complex profile with lower HRQoL; Type C demonstrated the highest rates of severe malnutrition and greater support needs.

Three distinct clinical-nutritional profiles were identified among the patient groups analyzed in the study. Group A was primarily made up of persons with pancreatitis (58.5%), admitted through medical services (97.6%). Most were non-malnourished (74.4%), with only 7.2% presenting severe malnutrition. A total of 34.8% were aged 40–59 years and they had low comorbidity (Charlson Index < 3, 86%), and no prior chemotherapy (90.8%). Functional and HRQoL indicators were favorable, with 57% scoring >92 on EuroQoL and 60.4% attaining 100 on the Barthel Index. Group B comprised mainly persons with CRC (76.1%), with a large proportion having undergone surgical intervention (91.4%). Severe malnutrition affected 10.2% of this group. Age distribution showed 23.4% between 40 and 59 years and 28% over 80 years, with a predominance of males (67%). Comorbidity varied, with 44.7% having three conditions and 55.3% none. Most had no prior chemotherapy (78.2%), and surgical complications were infrequent (13.7%). Hospital stays were short (<8 days among 83.2%), though 12.7% had EuroQoL scores of below 70. Group C included 30% of persons diagnosed with IBD, with 75.8% admitted through medical services; women accounted for 51.6% of the total. Their nutritional status was more compromised, with 26.8% showing severe malnutrition. Most had no prior chemotherapy (77.7%), and 68.2% had hospital stays of under 8 days. EurQoL scores ranged from 70 to 85 in 33.1% of patients, while 46.5% had Barthel scores of 60 to 100. Notably, 79.6% required or received social support (Figure 1).

### 3.4. Multivariable Model for HRQoL Variables of Persons with Cancer One Year After Hospitalization

Multilevel linear models revealed a significant association between baseline scores and better outcomes across all functional and HRQoL measures. The baseline Charlson Comorbidity Index score was negatively associated with EuroQoL (*p* < 0.05), SF-36 Mental Health (*p* < 0.05), and several physical domains. Polypharmacy was consistently associated with poorer outcomes, with significant negative associations across EuroQoL (*p* < 0.001), SF-36 Social Functioning (*p* < 0.001), and General Health (*p* < 0.001). Being female was linked to lower changes in in Barthel, Physical Functioning, Physical Role, and General Health (*p* < 0.05, respectively). Age was inversely related to Barthel and physical domains of SF-36 (*p* < 0.05, respectively). Having undergone surgery was significantly associated with worse Mental Health, Vitality, and Body Pain (*p* < 0.05, *p* < 0.05, and *p* < 0.001, respectively). No significant associations were identified between GLIM status and any of the outcomes (Table 3).

### 3.5. Multivariable Model for HRQoL Variables of Persons with Digestive Diseases One Year After Hospitalization

Multilevel linear models showed a significant association between baseline scores and all functional and HRQoL outcomes. Being female was significantly associated with lower EuroQoL, Barthel (*p* < 0.05, and *p* < 0.001, respectively), and multiple SF-36 dimensions, including Mental Health (*p* < 0.001) and Vitality (*p* < 0.001). Polypharmacy was consistently linked to poorer outcomes, particularly in Social Functioning (*p* < 0.001) and Body Pain (*p* < 0.001). The baseline Charlson Comorbidity Index score negatively impacted in Barthel and Emotional Role. Length of admission was inversely associated with Physical Role (*p* < 0.05) and Body Pain (*p* < 0.05). Persons with pancreatitis reported significantly better General Health (*p* < 0.05) than those with colitis. GLIM status displayed limited associations, only significant in the case of Vitality and Body Pain (*p* < 0.05 in both) (Table 4).

### 3.6. Multivariable Model for HRQoL Variables of All Persons One Year After Hospitalization

When all patients were analyzed together, baseline HRQoL, comorbidity, gender, and polypharmacy emerged as the strongest predictors of one-year outcomes, whereas GLIM-defined malnutrition showed limited independent effects. In addition, interaction analyses between gastrointestinal cancers and other digestive diseases did not reveal significant differential effects for most predictors (Table S3).

## 4. Discussion

This study offers a comprehensive analysis of HRQoL in a cohort of 560 persons with oncological (n = 284) and digestive pathologies (n = 276), stratified by nutritional status at hospital admission and followed up after one year. The findings reveal distinct clinical-nutritional profiles, clinical meaningful subgroups and factors associated with HRQoL one year after admission. However, the results show that GLIM-defined malnutrition at baseline does not independently predict HRQoL at one year.

In the study, 34.82% of hospitalized patients were found to be (moderately or severely) malnourished on admission. This result is slightly higher than that found in recent studies in Spain, which observed malnutrition among between 24.4% [10] and 29.7% [9] of hospitalized patients. Our data confirm that advanced age, high comorbidity, medical admission, and poor baseline physical status contribute to a more vulnerable clinical profile, consistent with observed mortality. This result confirms a strong association between physical baseline status and higher mortality [23] and suggests the need for early screening strategies to identify risk profiles.

### 4.1. Clinical Profiles

The use of MCA in this study proved to be a powerful exploratory tool for identifying three latent clinical-nutritional profiles, which would not have been evident through traditional regression models alone. Our findings highlight notable differences in nutritional status and HRQoL across participant groups with oncological and digestive diseases. Group A, composed primarily of persons with pancreatitis, showed the most favorable outcomes. The results are in keeping with previous studies suggesting that good nutritional status in acute digestive conditions may help preserve functional status and mitigate declines in HRQoL [24]. In contrast, Group B—predominantly persons with CRC undergoing surgical intervention—presented a more complex clinical profile. Prior research has shown that surgical stress, comorbidities, and advanced age are associated with poorer postoperative recovery and HRQoL outcomes in oncological populations [6]. These findings underscore the need for targeted nutritional and rehabilitative strategies in surgical oncology settings, where functional recovery and HRQoL may be at greater risk. Group C, which above all included persons with IBD, exhibited the highest rate of severe malnutrition and a substantial need for social support. These results reflect the chronic and relapsing nature of digestive pathologies; for this reason, previous studies have emphasized the multidimensional burden of IBD, malnutrition, and social isolation, all of which negatively impact HRQoL [25,26]. Overall, the MCA data reinforce the importance of incorporating nutritional assessment and support into long-term care pathways, particularly for persons with chronic digestive conditions and those undergoing oncological surgery.

### 4.2. Baseline, Follow-Up, and Changes in HRQoL and Nutritional Status

Baseline HRQoL scores varied significantly across malnutrition categories. Persons with CRC, severely malnourished, had significantly lower vitality, emotional role, and global health scores, indicating that nutritional status has a profound impact on psychological and physical well-being. Similar patterns were observed in persons with other cancers and IBD. These findings are consistent with previous studies on persons with gastrointestinal issues, in which the authors noted that a high prevalence of malnutrition was associated with lower physical and global HRQoL [27,28]. Interestingly, EuroQoL and Barthel Index scores were not found to be associated with nutritional groups, suggesting that global health perception and basic functional independence may be less sensitive to nutritional status at admission. The prevalence of malnutrition based on GLIM criteria on admission among persons with cancer was similar to that found in other studies [29,30]; in our study, the prevalence was 39.14% (81/207) among persons with CRC, including 22.71% (47/207) with moderate malnutrition and 16.43% (34/207) with severe malnutrition; similarly, in Liu et al. (2021), the figures were 40.1% (231/576) and 49.4% (278/563), respectively [31]. By pathology type, moderate malnutrition was most prevalent among persons with IBD (45.16%, 28/110), and least prevalent among persons with pancreatitis (13.86%, 23/166), while severe malnutrition was most prevalent (24.68%, 19/77) in persons with other cancers (esophageal, gastric, and pancreatic cancers). Similar figures were found in a study on persons with cancer, where the prevalence of malnutrition was also highest in persons with gastric cancer [30]. Our results on the nutritional support received by our malnourished patients are similar to those indicated elsewhere in the literature [4,6], with a small percentage of persons with malnutrition receiving nutritional support. At one-year follow-up, most HRQoL domains showed stabilization across all nutritional groups in all domains of HRQoL. Notably, differences observed at baseline between persons with and without malnutrition were not found to be associated with most HRQoL domains at one year. The change analysis showed that persons with severe malnutrition and oncological pathologies experienced significant improvements in mental, social, and pain aspects. One plausible explanation is that these persons often present with very poor baseline HRQoL, making them particularly responsive to early symptom control, nutritional support, and stabilization during hospitalization. Persons with pancreatitis and moderate malnutrition also showed improvements in these areas, including vitality, which may reflect the typically reversible inflammatory course of pancreatitis once acute symptoms are managed. Conversely, persons with pancreatitis and severe malnutrition showed declines in physical role and social functioning, indicating a divergent recovery pattern, which may indicate that, in the context of severe metabolic stress, recovery is slower and functional deterioration persists despite clinical improvement. However, these interpretations should be viewed cautiously given the very small number of persons in this subgroup.

### 4.3. Multivariate Analysis: Factors Associated to HRQoL One Year After Admission

Multivariate models confirmed that baseline health status was a robust indicator of HRQoL across both cancer and digestive populations. This is consistent with previous findings [32,33], which demonstrated that baseline HRQoL data appear to provide the most reliable prognostic indicators of survival and quality of life in persons with cancer. Gender differences were observed consistently, with female participants reporting lower HRQoL scores in both diagnostic groups. While the evidence on gender as a predictor was contradictory [32], our results are in line with those of other studies which reported differences in HRQoL perception by gender [34,35,36,37]. Polypharmacy emerged as a significant negative indicator of HRQoL, particularly in the physical and social domains. This is consistent with the findings of other studies [38,39], which highlighted the detrimental impact of polypharmacy on patients’ quality of life. A higher Charlson Comorbidity Index score was also negatively associated with HRQoL, especially among persons with cancer, supporting the results of several studies [40,41] that found that comorbidity burden significantly reduces functional outcomes in oncology. Interestingly, while the aforementioned variables might have influenced the evolution during the follow-up period, GLIM-defined malnutrition was not found to be a potential factor related to HRQoL. This contrasts with previous studies [12] showing that malnutrition diagnosed by GLIM criteria was an effective predictor of quality of life in older persons with cancer. This discrepancy may reflect differences in age distribution, which influence the predictive value of GLIM criteria, since previous studies focused on older populations, where malnutrition has a greater prognostic weight [12]. Another explication could be that the impact of baseline malnutrition, although clinically relevant in acute phases, may be attenuated over time, either because nutritional status improves with follow-up care or because long-term HRQoL becomes increasingly influenced by chronic symptom burden, disease activity, psychological well-being, and treatment complications. Despite this, an assessment of nutritional status and consequent identification of persons at risk of being malnourished or severely malnourished is necessary for the potential introduction of appropriate nutritional treatment [16].

Moreover, surgical intervention showed contrasting effects on HRQoL across patient groups. In persons with cancer, it was associated with lower scores in mental health, vitality, and higher pain at follow-up, suggesting a more burdensome recovery process or adverse effects of cancer therapy, whereas persons with digestive diseases who underwent surgery reported improved physical function at the one-year follow-up, possibly reflecting improved disease control or functional restoration post-surgery [42]. These differences highlight the need to consider disease context and recovery expectations when evaluating surgical impact on quality of life. These findings also provide us with information that can be used to provide future oncological patients undergoing surgery with information on postoperative changes that may occur in their HRQoL. Also, length of hospital stay was negatively associated with HRQoL among persons with digestive diseases, particularly in physical domains such as body pain and physical role, but not among persons with cancer. This discrepancy may be attributed to the acute and reactive nature of digestive pathologies, where hospital stay directly reflects functional status [43]. By contrast, among persons with cancer, hospital stay is influenced by multiple factors not necessarily related to immediate severity, such as programmed treatments. Finally, with regard to the type of pathology, pancreatitis disease was associated with better general health, possibly reflecting the small proportion of malnourished persons in this group (16.26%).

In summary, our findings are largely consistent with the existing literature, reinforcing the importance of baseline health, gender, comorbidity, and medication burden in shaping HRQoL. However, the limited role of nutritional status in our models suggests the need for further research to refine assessment tools and explore potential mediators.

### 4.4. Strengths and Clinical Implications

These findings highlight the added value of MCA in revealing complex interdependencies between clinical and social determinants of health and supporting its inclusion in multidimensional patient assessments and care planning. These findings have several clinical implications. First, they highlight the need for systematic nutritional screening and timely intervention, particularly among persons with cancer with a high prevalence of malnutrition. However, nutritional support alone may not be sufficient to improve long-term HRQoL and should be incorporated into a broader, multidisciplinary approach that includes psychosocial support, comorbidity management, and rehabilitation. Second, baseline HRQoL assessment should be incorporated into routine clinical practice, since it is a strong predictor of recovery and may help identify persons at risk of poor outcomes. Third, special attention should be given to female participants, older adults, and those with high rates of comorbidity or polypharmacy, as these factors are consistent in predicting worse trajectories. Finally, in advanced gastrointestinal cancers and diseases, gastrointestinal symptoms and associated malnutrition have often progressed, reducing QOL before treatment. In patients treated effectively, these symptoms are likely to improve after hospitalization. This is consistent with our longitudinal findings, where patients with more compromised baseline profiles—such as those with severe malnutrition—showed some of the largest improvements in specific SF-36 domains over 12 months, despite not fully closing the gap with better nourished patients. These results support the notion that timely, integrated oncological and nutritional care can translate into meaningful gains in perceived health and functioning, even in advanced disease.

### 4.5. Limitations

This study has some limitations. One involves the attrition of participants over time. This is partially evidenced by the high rate of non-responses at follow-up (39.52%). Additionally, any research focusing on quality of life must contend with the inherent constraint that outcomes can only be assessed among individuals who are still alive at the time of follow-up. This is particularly relevant in studies involving persons affected by malnutrition, a condition associated with elevated mortality rates. Notably, persons for whom follow-up data were unavailable—either due to decease or lack of response—had the lowest baseline HRQoL scores. It should also be taken into account that our results may be biased since we lost those participants who died—they generally had poorer baseline health that responders—as well as those who did not complete the follow-up, who, despite being younger that the responders, also had worse baseline scores in various SF-36 domains; that is to say, we lost those who were worse at baseline, leaving us with a healthier group. It is also essential to take into account that this study was conducted during the COVID-19 pandemic, under atypical clinical circumstances, and with significant hospital restrictions that limited direct contact with patients. The findings may also have been influenced by the relatively long interval between baseline and follow-up assessments, especially in a population with severe diseases who may have experienced various adverse events during that time. Additionally, the small sample size of some subgroups, particularly severely malnourished persons with pancreatitis, may limit the generalizability of findings.

### 4.6. Conclusions

This study therefore seeks to provide a comprehensive, one-year analysis of HRQoL, and identify factors that influence HRQoL one year after admission in a diverse cohort of persons with oncological and chronic digestive diseases, stratified by nutritional status at hospital admission. Across both populations, the results show that GLIM-defined malnutrition at baseline does not independently predict HRQoL at one year among participants who survived and completed follow-up. Since patients with poorer baseline nutritional status and HRQoL were more likely to die or be lost to follow-up, these results should be interpreted as applying to the responder cohort rather than to the entire population initially enrolled. Additionally, to identify clinically meaningful subgroups among hospitalized persons, multiple correspondence analysis (MCA) was used to clarify the multifactorial nature of HRQoL and identify targets for more effective and personalized care.

## Figures and Tables

**Table 1:** Sociodemographic, clinical, and nutritional data at admission.

			GLIM	
	Total (n = 560)	No Malnutrition ^1^ (n = 365)	Moderate Malnutrition ^2^ (n = 118)	Severe Malnutrition ^3^ (n = 77)
*Sociodemographic and clinical data at admission*				
Age, years *	63.4 ± 16.2	63.0 ± 15.5 ^2^	66.4 ± 16.5 ^1,3^	60.6 ± 18.4 ^2^
Gender (female)	220 (39.3)	137 (37.5)	54 (45.8)	29 (37.7)
Pathology		^2,3^	^1,3^	^1,2^
CRC *	207 (36.96)	126 (34.5)	47 (39.8)	34 (44.2)
Other cancers *	77 (13.75)	38 (10.4)	20 (17.0)	19 (24.7)
IBD *	110 (19.64)	62 (17.0)	28 (23.7)	20 (26.0)
Pancreatitis *	166 (29.64)	139 (38.1)	23 (19.5)	4 (5.2)
Charlson Comorbidity Index ^†^	2.0 (0.0–3.0)	2.0 (0.0–3.0)	2.0 (1.0–3.0)	2.0 (0.0–3.0)
Number of drugs prescribed ^†^	4.0 (2.0–7.0)	4.0 (2.0–7.0)	4.0 (2.0–7.0)	3.0 (1.0–6.0)
Type of admission				
Medical	337 (60.2)	229 (62.7)	63 (53.4)	45 (58.4)
Surgical	223 (39.8)	136 (37.3)	55 (46.6)	32 (41.6)
Surgery intervention at admission	233 (41.6)	143 (39.2)	55 (46.6)	35 (45.5)
Days of admission ^†^	6.0 (4.0–8.0)	6.0 (4.0–8.0) ^2^	7.0 (5.0–9.0) ^1^	7.0 (4.0–8.0)
Infectious complications	16 (2.9)	11 (3.0)	3 (2.5)	2 (2.6)
*Anthropometric and nutritional data*				
Handgrip strength at admission *	27.4 ± 11.7	28.3 ± 12.2	25.2 ± 11.2	26.4 ± 9.7
Calf at admission *	35.8 ± 3.7	36.7 ± 3.72 ^3^	34.4 ± 3.01	33.5 ± 3.3 ^1^
Weight at admission *	72.6 ± 16.2	77.2 ± 16.02 ^3^	66.0 ± 12.21 ^3^	60.6 ± 12.5 ^1,2^
BMI at admission *	26.1 ± 5.3	27.7 ± 5.22 ^3^	24.0 ± 3.81 ^3^	21.4 ± 3.7 ^1,2^
Readmission at 90 days	59 (10.5)	35 (9.6) ^3^	9 (7.6) ^3^	15 (19.5) ^1,2^
Nutritional support				
General advice	15 (2.7)	7 (1.9) ^3^	2 (1.7) ^3^	6 (7.8) ^1,2^
Diet enrichment	1 (0.2)	0 (0.0) ^3^	0 (0.0)	1 (1.3) ^1^
ONS	20 (3.6)	10 (2.7) ^3^	2 (1.7) ^3^	8 (10.4) ^1,2^
EN	6 (1.1)	2 (0.5) ^3^	1 (0.8)	3 (3.9) ^1^
NGT/NYS	4 (0.7)	2 (0.5)	0 (0.0)	2 (2.6)
Gastrojejunostomy	2 (0.4)	0 (0.0)	1 (0.9)	1 (1.3)
PN	11 (2.0)	6 (1.6)	2 (1.7)	3 (3.9)
Medication	24 (4.3)	12 (3.3) ^3^	2 (1.7) ^3^	10 (13.0) ^1,2^

Note. * Mean ± standard deviation. ^†^ Median (IQR (interquartile range)). CRC: Colorectal cancer; IBD: Inflammatory bowel disease; Other cancers: esophageal, gastric, and pancreatic cancer. BMI: Body Mass Index; ONS: oral nutritional supplements; EN: enteral nutrition; NGT/NYS: nasogastric, or nasojejunal nutrition; PN: parenteral nutrition. Values with superscripts represent statistically significant differences between groups (^1^: no malnutrition; ^2^: moderate malnutrition; ^3^: severe malnutrition) at *p* < 0.05.

**Table 2:** Health-related quality of life baseline, one-year follow-up and change data of patients with colorectal cancer, other cancers, inflammatory bowel disease and pancreatitis, according to malnutrition group through GLIM criteria.

		Baseline		One Year Follow-Up				Change	
	Non-Malnourished ^1^ (n = 365)	Moderate Malnutrition ^2^ (n = 118)	Severe Malnutrition ^3^ (n = 77)	Non-Malnourished ^1^ (n = 365)	Moderate Malnutrition ^2^ (n = 118)	Severe Malnutrition ^3^ (n = 77)	Non-Malnourished ^1^ (n = 365)	Moderate Malnutrition ^2^ (n = 118)	Severe Malnutrition ^3^ (n = 77)
	Mean (sd)	Mean (sd)	Mean (sd)	Mean (sd)	Mean (sd)	Mean (sd)	Median (IQR)	Median (IQR)	Median (IQR)
**Barthel**									
CRC	95.1 ± 9.6	94.4 ± 13.5	95.4 ± 6.6	93.9 ± 9.8	92.2 ± 11.7	95.5 ± 8.4	0.0 (−5.0–0.0)	0.0 (−10.0–0.0)	0.0 (0.0–0.0)
Other cancers	97.1 ± 5.0	91.8 ± 11.3	93.8 ± 11.8	93.5 ± 11.2	93.1 ± 8.8	95.5 ± 8.1	0.0 (−7.5–0.0)	0.0 (−5.0–5.0)	0.0 (−2.5–7.5)
IBD	96.3 ± 5.2	93.5 ± 7.9	95.0 ± 6.7	95.7 ± 5.9	95.0 ± 11.5	95.8 ± 5.8	0.0 (−5.0–0.0)	0.0 (0.0–5.0)	0.0 (0.0–0.0)
Pancreatitis	96.5 ± 7.7	94.5 ± 12.0	97.5 ± 2.9	94.9 ± 11.0	92.5 ± 11.8	96.3 ± 4.8	0.0 (0.0–0.0)	0.0 (−5.0–0.0)	0.0 (−2.5–0.0)
**EuroQoL**									
CRC	0.8 ± 0.2	0.9 ± 0.2	0.8 ± 0.2	0.8 ± 0.2	0.9 ± 0.2	0.8 ± 0.2	0.0 (−0.1–0.1)	0.0 (−0.1–0.2)	0.0 (−0.1–0.1)
Other cancers	0.8 ± 0.2	0.8 ± 0.2	0.8 ± 0.1	0.8 ± 0.2	0.8 ± 0.1	0.9 ± 0.1	0.0 (−0.1–0.1)	0.0 (0.0–0.1)	0.0 (−0.0–0.3)
IBD	0.8 ± 0.2	0.9 ± 0.1	0.8 ± 0.1	0.9 ± 0.2	0.9 ± 0.2	0.9 ± 0.1	0.0 (−0.0–0.1)	0.0 (−0.0–0.1)	0.1 (0.0–0.1)
Pancreatitis	0.8 ± 0.2	0.8 ± 0.2	0.8 ± 0.2	0.9 ± 0.2	0.9 ± 0.2	0.8 ± 0.1	0.0 (−0.0–0.1)	0.0 (−0.0–0.1)	0.0 (0.0–0.1)
**SF-36 MH**									
CRC	65.7 ± 15.8 ^3^	60.9 ± 16.9	54.9 ± 14.6 ^1^	64.9 ± 17.8	67.3 ± 16.8	61.3 ± 18.6	0.0 (−8.0–8.0) ^2,3^	4.0 (−4.0–16.0) ^1^	12.0 (−8.0–20.0) ^1^
Other cancers	62.7 ± 19.4	62.7 ± 19.9	60.4 ± 19.8	64.0 ± 17.7	64.2 ± 21.7	71.8 ± 12.1	0.0 (−4.0–8.0)	4.0 (−12.0–12.0)	12.0 (−4.0–28.0)
IBD	59.5 ± 16.9	61.7 ± 14.5	57.5 ± 18.2	65.0 ± 16.4	66.2 ± 13.9	65.4 ± 10.3	2.0 (−4.0–16.0)	12.0 (−4.0–20.0)	4.0 (−8.0–12.0)
Pancreatitis	65.8 ± 18.1	56.0 ± 21.4	54.0 ± 28.0	64.9 ± 17.4	64.2 ± 16.6	51.0 ± 12.4	0.0 (−12.0–8.0) ^2^	4.0 (0.0–18.0) ^1^	−4.0 (−16.0–10.0)
**SF-36** **PF**									
CRC	68.3 ± 30.6	61.1 ± 32.5	67.7 ± 25.5	68.4 ± 30.2	65.1 ± 32.8	62.1 ± 27.6	0.0 (−15.0–10.0)	0.0 (−20.0–10.0)	−5.0 (−15.0–5.0)
Other cancers	66.3 ± 28.2	61.1 ± 30.9	54.7 ± 28.2	62.8 ± 31.6	67.1 ± 24.9	73.9 ± 18.1	−5.0 (−20.0–5.0) ^2,3^	5.0 (0.0–20.0) ^1^	10.0 (0.0–55.0) ^1^
IBD	79.3 ± 24.3	71.1 ± 26.4	77.4 ± 25.1	81.5 ± 22.2	74.5 ± 30.7	83.5 ± 22.2	0.0 (−5.0–10.0)	5.0 (−10.0–10.0)	0.0 (−5.0–15.0)
Pancreatitis	74.0 ± 26.8	71.0 ± 28.6	81.3 ± 19.3	71.7 ± 29.8	74.8 ± 29.6	80.0 ± 22.7	0.0 (−10.0–5.0)	5.0 (−5.0–20.0)	−2.5 (−5.0–2.5)
**SF-36** **SF**									
CRC	75.6 ± 25.8	69.3 ± 35.4	63.3 ± 28.5	73.9 ± 29.4	77.5 ± 26.7	75.4 ± 29.9	0.0 (−25.0–25.0)	0.0 (−12.5–37.5)	12.5 (0.0–25.0)
Other cancers	71.8 ± 30.5 ^3^	63.2 ± 28.7	40.8 ± 43.3 ^1^	66.9 ± 30.2	69.7 ± 28.7	75.7 ± 23.4	0.0 (−25.0–12.5) ^3^	6.3 (0.0–25.0)	37.5 (0.0–62.5) ^1^
IBD	63.9 ± 28.3	67.1 ± 26.0	44.1 ± 35.4	72.7 ± 29.4	76.4 ± 25.1	78.1 ± 19.0	0.0 (−12.5–25.0) ^3^	12.5 (0.0–37.5)	25.0 (12.5–62.5) ^1^
Pancreatitis	74.4 ± 29.3	61.9 ± 32.9	75.0 ± 28.9	76.5 ± 28.5	79.5 ± 26.0	59.4 ± 15.7	0.0 (−12.5–12.5) ^2^	25.0 (0.0–37.5) ^1,3^	−18.8 (−31.3–0.0) ^2^
**SF-36 PR**									
CRC	70.5 ± 32.5	66.8 ± 34.9	57.0 ± 30.8	67.9 ± 33.2	64.0 ± 33.4	58.1 ± 37.6	0.0 (−25.0–12.5)	0.0 (−25.0–25.0)	0.0 (−18.8–6.3)
Other cancers	58.9 ± 35.2	51.6 ± 37.2	42.4 ± 39.9	54.4 ± 36.5	63.4 ± 31.7	64.9 ± 30.5	0.0 (−25.0–6.3) ^3^	18.8 (0.0–37.5)	21.9 (0.0–50.0) ^1^
IBD	59.2 ± 32.8 ^3^	60.9 ± 32.9 ^3^	38.5 ± 29.7 ^1,2^	71.8 ± 32.3	71.5 ± 33.4	68.1 ± 36.3	0.0 (−12.5–37.5)	9.4 (−12.5–25.0)	25.0 (12.5–56.3)
Pancreatitis	73.2 ± 29.9 ^2^	53.0 ± 34.5 ^1^	71.9 ± 29.1	71.4 ± 32.6	77.3 ± 32.0	59.4 ± 27.7	0.0 (−12.5–12.5) ^2^	21.9 (0.0–62.5) ^1,3^	−12.5 (−21.9–3.1) ^2^
**SF-36 ER**									
CRC	83.9 ± 23.9 ^3^	89.3 ± 19.2 ^3^	71.5 ± 28.2 ^1,2^	78.6 ± 28.6	74.4 ± 30.0	72.5 ± 30.9	0.0 (−16.7–0.0)	0.0 (−33.3–0.0)	0.0 (−4.2–12.5)
Other cancers	84.5 ± 22.9	76.3 ± 25.0	78.1 ± 31.1	78.0 ± 29.6	71.8 ± 29.4	87.5 ± 20.1	0.0 (−25.0–0.0)	0.0 (−25.0–16.7)	0.0 (0.0–33.3)
IBD	79.9 ± 26.2	83.0 ± 23.7	76.8 ± 30.4	81.6 ± 24.3	83.3 ± 25.3	86.7 ± 15.4	0.0 (−8.3–0.0)	0.0 (−16.7–0.0)	0.0 (0.0–25.0)
Pancreatitis	85.5 ± 22.0	87.7 ± 19.8	87.5 ± 25.0	79.8 ± 25.9	82.5 ± 27.1	66.7 ± 26.4	0.0 (−25.0–8.3)	0.0 (−8.3–0.0)	−16.7 (−37.5–4.2)
**SF-36 Vitality**									
CRC	58.8 ± 20.8 ^3^	54.2 ± 19.9	47.0 ± 19.6 ^1^	56.8 ± 20.1	57.7 ± 20.5	52.2 ± 16.9	0.0 (−10.0–10.0)	0.0 (−15.0–20.0)	5.0 (−10.0–20.0)
Other cancers	52.6 ± 25.0	54.2 ± 23.2	41.1 ± 23.2	53.1 ± 22.7	54.7 ± 21.5	55.5 ± 15.6	0.0 (−10.0–15.0)	5.0 (−5.0–15.0)	10.0 (0.0–35.0)
IBD	50.1 ± 18.6	49.4 ± 18.1	40.0 ± 20.4	53.5 ± 18.9	57.9 ± 20.9	54.3 ± 15.1	0.0 (−10.0–15.0)	10.0 (−15.0–25.0)	20.0 (0.0–30.0)
Pancreatitis	56.5 ± 19.0	46.2 ± 19.7	52.5 ± 29.0	56.6 ± 21.0	58.6 ± 18.3	41.7 ± 24.7	0.0 (−10.0–15.0) ^2^	5.0 (0.0–30.0) ^1^	5.0 (−10.0–10.0)
**SF-36 BP**									
CRC	71.8 ± 30.2	73.9 ± 29.5	60.3 ± 29.9	67.9 ± 29.7	70.9 ± 30.9	65.7 ± 31.3	0.0 (−22.5–10.0)	0.0 (−22.5–20.0)	0.0 (−12.5–12.5)
Other cancers	61.3 ± 37.7	57.2 ± 34.6	42.2 ± 37.8	65.8 ± 30.6	69.7 ± 30.0	70.5 ± 18.6	0.0 (0.0–22.5) ^3^	6.3 (−10.0–22.5)	32.5 (0.0–55.0) ^1^
IBD	52.1 ± 31.4	55.6 ± 35.2	42.1 ± 30.1	67.6 ± 30.6	73.5 ± 27.7	73.8 ± 28.1	10.0 (−10.0–42.5)	21.3 (−10.0–45.0)	32.5 (0.0–67.5)
Pancreatitis	53.1 ± 32.8	42.7 ± 22.9	60.0 ± 39.6	64.0 ± 29.9	73.4 ± 30.7	52.5 ± 33.8	2.5 (−12.5–32.5) ^2^	32.5 (2.5–45.0) ^1,3^	−10.0 (−16.3–1.3) ^2^
**SF-36 GH**									
CRC	58.6 ± 17.7 ^3^	59.4 ± 19.5 ^3^	49.8 ± 18.6 ^1,2^	54.4 ± 22.0	56.1 ± 21.2	47.4 ± 20.0	−5.0 (−15.0–5.0)	0.0 (−20.0–10.0)	−5.0 (−15.0–10.0)
Other cancers	59.6 ± 19.3	55.8 ± 15.5	53.2 ± 18.3	51.1 ± 22.7	49.7 ± 22.1	56.3 ± 17.9	−5.0 (−15.0–0.0)	−5.0 (−15.0–12.5)	5.0 (−20.0–20.0)
IBD	41.7 ± 20.2	40.7 ± 14.2	46.3 ± 22.4	45.6 ± 20.0	44.6 ± 21.0	48.8 ± 23.2	5.0 (−5.0–10.0)	5.0 (−12.5–12.5)	0.0 (−15.0–10.0)
Pancreatitis	55.0 ± 19.9	50.0 ± 15.6	47.5 ± 15.5	55.6 ± 24.0	59.1 ± 15.2	43.8 ± 10.3	0.0 (−10.0–15.0)	10.0 (0.0–15.0)	−5.0 (−10.0–2.5)

Note. Values with superscripts represent statistically significant differences between groups (^1^: no malnutrition; ^2^: moderate malnutrition; ^3^: severe malnutrition) at *p* < 0.05. IQR: interquartile range. CRC: Colorectal cancer; IBD: Inflammatory bowel disease; Other cancers: esophageal, gastric, and pancreatic cancer. MH: Mental health; PF: Physical functioning; SF: Social functioning; PR: Physical role; ER: Emotional role; BP: Body pain; GH: General health.

**Table 3:** Multivariable multilevel linear models for functional status and health-related quality of life outcomes for digestive cancer patients one year after hospitalization.

Functional Status and Health-Related Quality of Life Outcomes										
	EuroQoL	Barthel	SF36-MH	SF36-SF	SF36-PF	SF36-PR	SF36-ER	SF36-VT	SF36-BP	SF36-GH
	Beta (95% CI ^1^)	Beta (95% CI ^1^)	Beta (95% CI ^1^)	Beta (95% CI ^1^)	Beta (95% CI ^1^)	Beta (95% CI ^1^)	Beta (95% CI ^1^)	Beta (95% CI ^1^)	Beta (95% CI ^1^)	Beta (95% CI ^1^)
**Variables at admission**										
**Baseline domain**	0.24 ** (0.14, 0.33)	0.19 * (0.01, 0.37)	0.45 ** (0.33, 0.56)	0.26 ** (0.15, 0.37)	0.38 ** (0.27, 0.48)	0.36 ** (0.24, 0.47)	0.51 ** (0.37, 0.65)	0.50 ** (0.40, 0.60)	0.39 ** (0.28, 0.49)	0.49 ** (0.36, 0.62)
**GLIM**										
No malnutrition	Ref.	Ref.	Ref.	Ref.	Ref.	Ref.	Ref.	Ref.	Ref.	Ref.
Moderate malnutrition	0.02 (−0.03, 0.07)	0.48 (−2.6, 3.6)	2.1 (−2.6, 6.8)	3.0 (−5.1, 11)	3.2 (−4.0, 10)	4.4 (−5.0, 14)	−2.4 (−11, 5.9)	1.3 (−3.9, 6.5)	1.5 (−6.6, 9.6)	1.7 (−3.6, 7.1)
Severe malnutrition	0.02 (−0.04, 0.07)	3.1 (−0.25, 6.5)	2.1 (−3.0, 7.3)	7.2 (−1.5, 16)	−0.27 (−7.8, 7.3)	0.78 (−9.6, 11)	4.9 (−3.9, 14)	2.1 (−3.5, 7.7)	3.8 (−5.1, 13)	0.87 (−4.9, 6.7)
**Gender (female)**	-	−3.6 * (−6.2, −0.98)	-	-	−7.5 * (−14, −1.2)	−9.1 * (−17, −1.0)	-	-	-	−5.3 * (−9.8, −0.76)
**Age**	-	−0.15 * (−0.25, −0.04)	-	-	−0.57 ** (−0.81, −0.34)	−0.33 * (−0.61, −0.04)	−0.34 * (−0.60, −0.08)	-	-	-
**CCI**	−0.01 * (−0.03, 0.00)	-	−1.2 * (−2.4, −0.05)	-	−2.5 * (−4.3, −0.76)	-	-	-	−2.4 * (−4.3, −0.41)	−1.8 * (−3.2, −0.45)
**N° of prescribed drugs**	−0.01 ** (−0.02, −0.01)	−0.47 * (−0.88, −0.06)	−0.70 * (−1.3, −0.12)	−1.4 ** (−2.4, −0.47)	−1.3 * (−2.3, −0.41)	-	-	−0.69 * (−1.3, −0.05)	−1.4 ** (−2.4, −0.47)	−1.3 ** (−2.0, −0.63)
**Surgical intervention ^2^**	-	-	−4.9 * (−9.4, −0.34)	-	-	-	-	−5.3 * (−10, −0.18)	−11 ** (−19, −3.2)	-
**R^2^**	0.18	0.14	0.23	0.12	0.38	0.20	0.19	0.29	0.21	0.35

Note. * *p* < 0.05; ** *p* < 0.001; ns: not statistically significant. ^1^ CI = Confidence Interval; ^2^ Type of admission: surgical intervention. -: not included in the model. MH: Mental health; PF: Physical functioning; SF: Social functioning; PR: Physical role; ER: Emotional role; BP: Body pain; GH: General health. CCI: Charlson Comorbidity Index.

**Table 4:** Multivariable multilevel linear models for functional status and health-related quality of life outcomes for digestive chronic patients one year after hospitalization.

Functional Status and Health-Related Quality of Life Outcomes										
	EuroQoL	Barthel	SF36-MH	SF36-SF	SF36-PF	SF36-PR	SF36-ER	SF36-VT	SF36-BP	SF36-GH
	Beta (95%CI ^1^)	Beta (95% CI ^1^)	Beta (95% CI ^1^)	Beta (95% CI ^1^)	Beta (95% CI ^1^)	Beta (95% CI ^1^)	Beta (95% CI ^1^)	Beta (95% CI ^1^)	Beta (95% CI ^1^)	Beta (95% CI ^1^)
**Variables at admission**										
**Baseline domain**	0.40 ** (0.32, 0.49)	0.60 ** (0.46, 0.74)	0.39 ** (0.29, 0.49)	0.32 ** (0.22, 0.42)	0.66 ** (0.56, 0.75)	0.41 ** (0.30, 0.52)	0.32 ** (0.20, 0.45)	0.46 ** (0.35, 0.58)	0.22 ** (0.12, 0.33)	0.68 ** (0.57, 0.80)
**GLIM**										
No malnutrition	Ref.	Ref.	Ref.	Ref.	Ref.	Ref.	Ref.	Ref.	Ref.	Ref.
Moderate malnutrition	0.03 (0.02, 0.07)	0.31 (−2.4, 3.0)	3.0 (−1.8, 7.8)	7.1 (−1.0, 15)	5.6 (−0.22, 11)	7.2 (−2.0, 16)	3.2 (−4.4, 11)	6.6 * (0.95, 12)	11 * (1.8, 20)	1.9 (−4.0, 7.8)
Severe malnutrition	−0.02 (−0.08, 0.04)	0.63 (−2.9, 4.2)	−0.78 (−7.0, 5.4)	3.5 (−7.4, 14)	0.88 (−7.1, 8.9)	−0.20 (−13, 12)	3.0 (−7.1, 13)	1.5 (−6.4, 9.3)	4.6 (−7.4, 17)	−2.7 (−11, 5.5)
**Gender (female)**	−0.04 * (−0.08, −0.01)	−3.3 ** (−5.4, −1.2)	−6.4 ** (−9.9, −2.8)	−8.4 ** (−14, −2.4)	-	-	−8.6 ** (−15, −2.7)	−6.9 ** (−11, −2.6)	−7.3 * (−14, −0.56)	
**Age**	-	-	-	-	-	−0.40 ** (−0.61, −0.18)	-	-	-	-
**CCI**	-	−1.3 * (−2.3, −0.24)	-	-	-	-	−3.1 * (−5.7, −0.53)	-	-	-
**N° of prescribed drugs**	−0.01 ** (−0.02, −0.01)	−0.41 * (−0.74, −0.07)	−0.70 ** (−1.2, −0.19)	−2.0 ** (−2.8, −1.1)	−1.1 ** (−1.8, −0.31)	−1.5 (−2.6, −0.38)	-	−1.1 ** (−1.7, −0.45)	−2.6 ** (−3.5, −1.6)	-
**Surgical intervention ^2^**	-	-	-	-	11 * (1.2, 20)	-	-	-	-	-
**Type of pathology**										
Colitis	-	-	-	-	-	-	-	-	-	Ref.
Crohn	-	-	-	-	-	-	-	-	-	1.0 (−6.1, 8.1)
Pancreatitis	-	-	-	-	-	-	-	-	-	7.1 * (0.65, 13)
**Days of admission**	-	-	-	-	-	−0.79 * (−1.5, −0.08)	-	-	−0.90 * (−1.6, −0.21)	-
**R^2^**	0.40	0.36	0.30	0.60	0.26	0.33	0.17	0.34	0.22	0.42

Note. * *p* < 0.05; ** *p* < 0.001; ns: not statistically significant. ^1^ CI = Confidence Interval; ^2^ Type of admission: surgical intervention. -: not included in the model. MH: Mental health; PF: Physical functioning; SF: Social functioning; PR: Physical role; ER: Emotional role; BP: Body pain; GH: General health. CCI: Charlson Comorbidity Index.

## Data Availability

The datasets generated during and/or analyzed in the current study are available from the corresponding author on reasonable request.

## Supplementary Materials

The following supporting information can be downloaded at: https://www.mdpi.com/article/10.3390/jcm15103596/s1, Figure S1: Flow-chart of the recruitment and follow-up process for the sample of patients with and without malnutrition. Table S1: Comparison of type of admitted patient according to response. Table S2: Subgroups of malnourished patients according to pathology. Table S3: Multivariable multilevel linear models for functional status and health-related quality of life outcomes for all the patients one year after hospitalization.

## Abbreviations

BMI	Body Mass Index
CEIC	Clinical Research Ethics Committee
CRC	Colorectal Cancer
GLIM	Global Leadership Initiative on Malnutrition
HRQoL	Health-Related Quality of Life
IBD	Inflammatory Bowel Disease
MCA	Multiple Correspondence Analysis
MUST	Malnutrition Universal Screening Tool
VAS	Visual Analogue Scale
