# Supplementary material for: Health-Related Quality of Life Changes in Patients with Digestive Cancers and Chronic Digestive Diseases: A Prospective, Multicenter Study
Source: J Clin Med. 2026 May 8;15(10):3596. doi: 10.3390/jcm15103596 (PMC13207875; doi:10.3390/jcm15103596)
Supplement: Supplementary file 1 [file jcm-15-03596-s001.zip › jcm-4249936-supplementary.pdf]

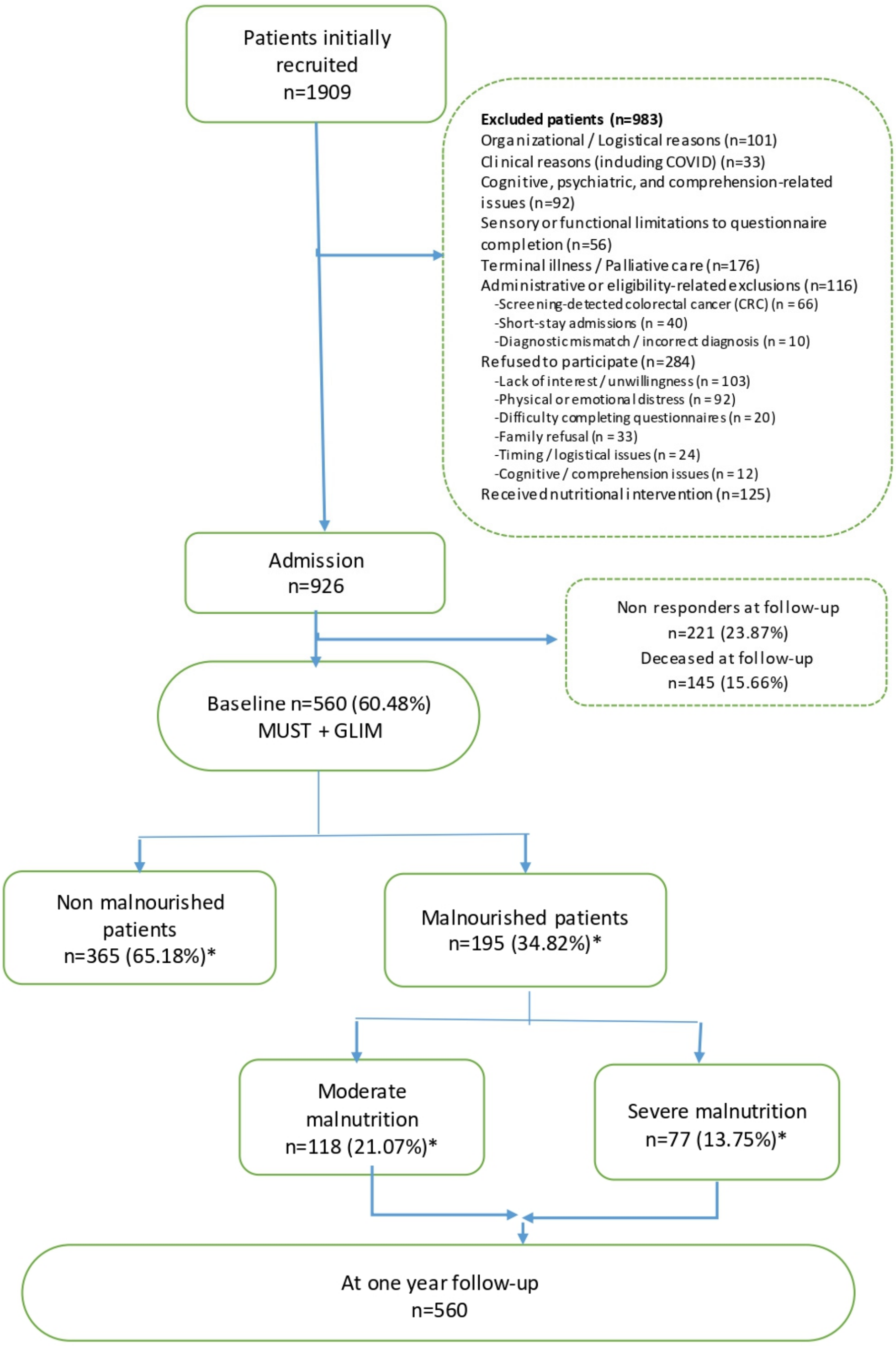

Figure S1: Flow-chart of the recruitment and follow-up process for the sample of patients with and without malnutrition

Table S1. Comparison of type of admitted patient according to response.

**Variables**	**Total (n=926)**	**Non responders[1] (n=221)**	**Responders[2] (n=560)**	**Deceased[3] (n=145)**	**P value**
Sociodemographic and clinical data at admission					
Age*	62.6±16.4	56.0±16.8[2,3]	63.4±16.2[1,3]	69.3±12.5[1,2]	**<.0001**
Charlson Index †	2.0 (0.0–3.0)	2.0 (0.0–2.0)[3]	2.0 (0.0–3.0)[3]	6.0 (3.0–7.0)[1,2]	**<.0001**
Number of drugs †	4.0 (2.0–7.0)	3.0 (1.0–6.0)[3]	4.0 (2.0–7.0)[3]	6.0 (3.0–9.0)[1,2]	**<.0001**
Main pathology		[3]	[3]	[1,2]	**<.0001**
CRC	179 (19.3)	26 (11.8)	77 (13.8)	76 (52.4)	
Other cancers	340 (36.7)	69 (31.2)	207 (37.0)	64 (44.1)	
IBD	156 (16.8)	45 (20.4)	110 (19.6)	1 (0.7)	
Pancreatitis	251 (27.1)	81 (36.7)	166 (29.6)	4 (2.8)	
Gender (female)	370 (40.0)	99 (44.8)	220 (39.3)	51 (35.2)	0.16
Type of admission		[3]	[1]	[1,2]	**<.0001**
Medical	603 (65.1)	145 (65.6)	337 (60.2)	121 (83.4)	
Surgical	323 (34.9)	76 (34.4)	223 (39.8)	24 (16.6)	
Surgery intervention	342 (36.9)	80 (36.2)[3]	233 (41.6)[3]	29 (20.0)[1,2]	**<.0001**
Anthropometric and nutritional data					
Handgrip strength at admission*	27.3±11.8	29.2±12.6[3]	27.4±11.7[3]	24.1±10.5[1,2]	**0.0009**
Weight at admission*	72.2±16.0	74.3±16.0[3]	72.6±16.2[3]	67.6±14.3[1,2]	**0.0002**
Calf at admission*	35.6±3.8	36.2±3.7[3]	35.8±3.7[3]	34.1±4.0[1,2]	**<.0001**
BMI at admission*	25.9±5.2	26.5±5.0[3]	26.1±5.3[3]	24.4±4.6[1,2]	**0.0002**
Days of admission*	7.1±4.7	7.2±5.0	7.0±4.4	7.1±5.1	0.74
Readmission at 90 days	155 (16.7)	29 (13.1)[3]	59 (10.5)[3]	67 (46.2)[1,2]	**<.0001**
GLIM at admission		[3]	[3]	[1,2]	**<.0001**
No malnutrition	573 (61.9)	151 (68.3)	365 (65.2)	57 (39.3)	
Moderate malnutrition	199 (21.5)	43 (19.5)	118 (21.1)	38 (26.2)	
Severe malnutrition	154 (16.6)	27 (12.2)	77 (13.8)	50 (34.5)	
Nutritional support					
General advice	26 (2.8)	8 (3.6)	15 (2.7)	3 (2.1)	0.65
Diet enrichment	3 (0.3)	1 (0.5)	1 (0.2)	1 (0.7)	0.58
Adaptation	1 (0.1)	0 (0.0)	0 (0.0)	1 (0.7)	0.07
ONS	33 (3.6)	9 (4.1)	20 (3.6)	4 (2.8)	0.80
EN	9 (1.0)	0 (0.0)	6 (1.1)	3 (2.1)	0.13
NGT/NYS	8 (0.9)	0 (0.0)[3]	4 (0.7)[3]	4 (2.8)[1,2]	**0.02**
Gastrojejunostomy	2 (0.2)	0 (0.0)	2 (0.4)	0 (0.0)	0.52
PN	17 (1.8)	4 (1.8)	11 (2.0)	2 (1.4)	0.90
Medication	38 (4.1)	6 (2.7)	24 (4.3)	8 (5.5)	0.39
Health-related Quality of Life questionnaires at baseline					
Barthel*	94.5±10.8	95.1±9.2[3]	95.5±8.8[3]	89.5±17.4[1,2]	**0.005**
EuroQoL*	0.8±0.2	0.8±0.2[3]	0.8±0.2[3]	0.7±0.2[1,2]	**<.0001**
SF36 Social Functioning*	64.4±32.8	61.2±33.5[2,3]	68.6±30.8[1,3]	52.4±35.8[1,2]	**<.0001**
SF36 Mental Health*	60.4±18.0	57.6±17.7[2]	62.5±17.7[1,3]	56.3±18.9[2]	**<.0001**
SF36 Emotional Role*	80.1±26.6	75.5±27.8[2]	83.0±24.2[1]	75.6±32.1	**0.002**
SF36 Physical Functioning*	66.6±30.4	69.4±30.9[3]	70.1±28.5[3]	49.3±31.4[1,2]	**<.0001**
SF36 Physical Role*	58.6±35.2	56.3±34.4[2,3]	63.9±33.7[1,3]	41.6±36.3[1,2]	**<.0001**
SF36 Vitality*	50.0±21.1	47.9±19.6[2,3]	53.2±20.8[1,3]	40.6±21.5[1,2]	**<.0001**
SF36 Body Pain*	54.4±34.0	51.1±33.9[2,3]	58.9±33.2[1,3]	41.9±33.3[1,2]	**<.0001**
SF36 General Health*	50.1±20.2	49.0±20.3[3]	53.3±19.6[3]	39.5±18.6[1,2]	**<.0001**

Note. *Mean ± standard deviation. †Median (IQR (interquartile range)). IBD: Inflammatory bowel disease; BMI: Body Mass Index. ONS: oral nutritional supplements; EN: enteral nutrition; NGT/NYS: nasogastric, or nasojejunal nutrition; PN: parenteral nutrition. Values with superscripts represent statistically significant differences between groups ([1]: non responders: patients who completed the HRQoL questionnaires at baseline but did not complete them at one year; [2]: responders: patients who completed the HRQoL questionnaires at baseline and at one year; [3]: deceased: patients who were defined as patients who completed the HRQoL questionnaires at baseline but died during the year) at p<0.05. P-values in bold represent statistically significant differences at p=0.05

Table S2. Subgroups of malnourished patients according to pathology

	**Non malnourished**[1] (n=365)	**Moderate malnutrition**[2] (n=118)	**Severe malnutrition**[3] (n=77)
	mean (sd)	mean (sd)	mean (sd)
**Pathology**	[2,3]	[1,3]	[1,2]
CRC*	126 (34.5)	47 (39.8)	34 (44.2)
Other cancers*	38 (10.4)	20 (17.0)	19 (24.7)
IBD*	62 (17.0)	28 (23.7)	20 (26.0)
Pancreatitis*	139 (38.1)	23 (19.5)	4 (5.2)

Note. CRC: Colorectal cancer; IBD: Inflammatory bowel disease; Other cancers: esophageal, gastric, and pancreatic cancer. *Mean ± standard deviation. Values with superscripts represent statistically significant differences between groups ([1]: no malnutrition; [2]: moderate malnutrition; [3]: severe malnutrition) at $p<0.05$.

Table S3. Multivariable multilevel linear models for functional status and health-related quality of life outcomes for all the patients one year after hospitalization

	**Functional status and health-related quality of life outcomes**									
	**EuroQoL**	**Barthel**	**SF36 - MH**	**SF36 - SF**	**SF36 - PF**	**SF36 - PR**	**SF36 - ER**	**SF36 - VT**	**SF36 - BP**	**SF36 - GH**
	**Beta (95%CI[1])**	**Beta (95% CI[1])**	**Beta (95% CI[1])**	**Beta (95% CI[1])**	**Beta (95% CI[1])**	**Beta (95% CI[1])**	**Beta (95% CI[1])**	**Beta (95% CI[1])**	**Beta (95% CI[1])**	**Beta (95% CI[1])**
**Variables**										
**Baseline domain**	0.31** (0.25, 0.38)	0.46** (0.35, 0.57)	0.43** (0.36, 0.51)	0.29** (0.21, 0.36)	0.48** (0.40, 0.55)	0.40** (0.33, 0.48)	0.41** (0.32, 0.51)	0.49** (0.41, 0.57)	0.32** (0.25, 0.40)	0.58** (0.49, 0.67)
**GLIM**										
No malnutrition	Ref.	Ref.	Ref.	Ref.	Ref.	Ref.	Ref.	Ref.	Ref.	Ref.
Moderate malnutrition	0.02 (-0.01, 0.06)	0.08 (-1.99, 2.15)	2.09 (-1.29, 5.47)	4.63 (-1.07, 10.33)	3.63 (-1.15, 8.40)	4.30 (-2.23, 10.84)	-1.19 (-6.79, 4.42)	3.86* (0.02, 7.70)	3.59 (-2.48, 9.65)	1.43 (-2.54, 5.39)
Severe malnutrition	0.00 (-0.04, 0.04)	1.76 (-0.74, 4.26)	1.68 (-2.20, 5.57)	6.17 (-0.60, 12.95)	0.17 (-5.38, 5.72)	-0.02 (-7.91, 7.86)	3.59 (-2.96, 10.13)	2.31 (-2.31, 6.93)	3.29 (-4.00, 10.57)	-0.56 (-5.20, 4.08)
**Pathology group (other digestive diseases)**	-0.01 (-0.07, 0.04)	-2.55 (-10.12, 5.02)	-7.78* (-14.30, -1.25)	6.13 (-2.68, 14.93)	-23.01* (-40.31, -5.71)	13.49 (-9.03, 36.02)	-0.46 (-7.56, 6.64)	-1.01 (-7.28, 5.25)	-5.82 (-16.60, 4.97)	-1.99 (-9.00, 5.03)
**Gender (female)**	-	-3.14* (-5.60, -0.68)	-	-	-5.56* (-11.08, -0.04)	-8.35* (-15.78, -0.92)	--		-	-5.08* (-9.56, -0.60)
**Age**	-	-0.15* (-0.24, -0.06)	-	-	-0.58** (-0.78, -0.37)	-0.32* (-0.59, -0.05)	-	-	-	-
**CCI**	-0.01* (-0.03, -0.00)	-	-1.11* (-2.21, -0.00)	-	-2.35* (-3.90, -0.80)	-	-	-	-2.24* (-4.21, -0.27)	-1.61* (-2.96, -0.26)

Variables										
**Surgical intervention**	-	-	-4.88 (-9.21, -0.55)	-	-	-	-	-5.12* (-10.08, -0.15)	-9.77* (-17.81, -1.73)	-
**Nº of prescribed drugs**	-0.01** (-0.02, -0.01)	-	-0.67* (-1.22, -0.12)	-1.36* (-2.30, -0.42)	-1.21* (-2.03, -0.38)	-	-1.76** (-2.69, -0.83)	-0.67* (-1.31, -0.04)	-	-1.17** (-1.84, -0.50)
**Pathology group: gender**	-	0.26 (-3.09, 3.61)	-	-	-0.16 (-7.76, 7.43)	2.29 (-8.11, 12.69)	-	-	-	-0.90 (-7.20, 5.39)
**Pathology group: CCI**	0.01 (-0.02, 0.03)	-	-0.05 (-2.17, 2.08)	-	1.10 (-2.00, 4.19)	-	-	-	-2.82 (-6.39, 0.75)	0.27 (-2.30, 2.83)
**Pathology group: days of admission**	-	-	-	-0.05 (-0.80, 0.70)	-	-	-	-	-	-
**Pathology group: age**	-	0.04 (-0.08, 0.15)	-	-	0.25 (-0.02, 0.52)	-0.16 (-0.50, 0.18)	-	-	-	-
**Pathology group: surgical intervention**	-	-	-	-	-	-	-	7.58 (-1.74, 16.90)	8.25 (-6.84, 23.33)	-
**Pathology group: nº of prescribed drugs**	-0.00 (-0.01, 0.01)	-	0.19 (-0.61, 1.00)	-0.61 (-1.90, 0.67)	-0.03 (-1.23, 1.17)	-	1.13 (-0.16, 2.42)	-0.34 (-1.21, 0.53)	-	0.31 (-0.65, 1.26)
**R^2**	0.26	0.21	0.25	0.16	0.46	0.24	0.17	0.30	0.14	0.37

Note. : Interaction. *p<0.05; **p<0.001. CI: Confidence Interval. CCI: Charlson Comorbidity Index. GI cancer: gastrointestinal cancer. Domains of SF-36: MH: Mental health; SF: Social functioning; PF: Physical functioning; PR: Physical role; ER: Emotional role; BP: Body pain; GH: General health.
